# Unlocking the potential of *in silico* approach in designing antibodies against SARS-CoV-2

**DOI:** 10.3389/fbinf.2025.1533983

**Published:** 2025-02-13

**Authors:** Tasshitra Subramaniam, Siti Aisyah Mualif, Weng Howe Chan, Khairul Bariyyah Abd Halim

**Affiliations:** ^1^ Biomedical Engineering and Health Sciences Department, Faculty of Electrical Engineering, Universiti Teknologi Malaysia, Johor Bahru, Johor, Malaysia; ^2^ Advanced Diagnostics and Progressive Human Care, Biomedical Engineering and Health Sciences Department, Faculty of Electrical Engineering, Universiti Teknologi Malaysia, Johor Bahru, Johor, Malaysia; ^3^ Faculty of Computing, Universiti Teknologi Malaysia, Johor Bahru, Johor, Malaysia; ^4^ Department of Biotechnology, Kulliyyah of Science, International Islamic University Malaysia, Kuantan, Pahang, Malaysia; ^5^ Research Unit for Bioinformatics and Computational Biology (RUBIC), Kulliyyah of Science, International Islamic University Malaysia, Kuantan, Pahang, Malaysia

**Keywords:** *in silico*, antibody, SARS-CoV-2, computational approach, bioinformatics, molecular dynamic simulation

## Abstract

Antibodies are naturally produced safeguarding proteins that the immune system generates to fight against invasive invaders. For centuries, they have been produced artificially and utilized to eradicate various infectious diseases. Given the ongoing threat posed by COVID-19 pandemics worldwide, antibodies have become one of the most promising treatments to prevent infection and save millions of lives. Currently, *in silico* techniques provide an innovative approach for developing antibodies, which significantly impacts the formulation of antibodies. These techniques develop antibodies with great specificity and potency against diseases such as SARS-CoV-2 by using computational tools and algorithms. Conventional methods for designing and developing antibodies are frequently costly and time-consuming. However, *in silico* approach offers a contemporary, effective, and economical paradigm for creating next-generation antibodies, especially in accordance with recent developments in bioinformatics. By utilizing multiple antibody databases and high-throughput approaches, a unique antibody construct can be designed *in silico*, facilitating accurate, reliable, and secure antibody development for human use. Compared to their traditionally developed equivalents, a large number of *in silico*-designed antibodies have advanced swiftly to clinical trials and became accessible sooner. This article helps researchers develop SARS-CoV-2 antibodies more quickly and affordably by giving them access to current information on computational approaches for antibody creation.

## 1 Introduction

The Coronavirus Disease 2019 (COVID-19) pandemic, which is caused by the SARS-CoV-2 virus (Severe Acute Respiratory Syndrome Coronavirus 2), has already claimed the lives of approximately 6.8 million people so far and as of right now, there is no effective therapy for COVID-19 as the virus is emerging (Infectious Diseases Society of America, 2024). To control the disease progression, various types of antiviral drugs (Al-Tawfiq et al., 2020; Beigel et al., 2020; Gordon et al., 2021; Arbel et al., 2022) and antibodies (Safarzadeh Kozani et al., 2022; Keam, 2022) were prescribed to COVID-19 patients. Although antibodies offer protection with higher specificity against SARS-CoV-2 than drugs but their limitations point out the challenges in developing sustainable antibodies in the phase of rapid viral evolution (Van Regenmortel, 2014).

COVID-19 therapeutic antibodies developed to target the key components of SARS-CoV-2, Spike (S) protein, which interacts with ACE2 receptor protein on the cells in the respiratory tract during viral invasion (Pizzato et al., 2022). However, continuous structural changes of S protein of SARS-CoV-2 caused by rapid mutations render the effectiveness of the therapeutic antibodies. The antibodies which have been approved by EUA to be prescribed for COVID-19 patients, lost the approval as the mAb is no longer effective against currently emerging SARS-CoV-2 (Orders, 2022; Keam, 2022).

In this case, *in silico* technology paves promising approaches to design antibodies with our desired formats and customize the residues that favor higher binding affinity and good developability in a shorter time frame (Wolf Pérez et al., 2022). According to Moore, the phrase “*in silico*” refers to computer-assisted experimental procedures used in modern research (Moore, 2021). The integration of *in silico* technology into pharmaceutical research, notably in antibody designing, offers a sustainable approach and complementary avenue to traditional experimental methods that facilitates efficient antibody discovery for SARS-CoV-2 while conserving time and resources (Jabalia et al., 2021; Shaker et al., 2021; Ivanov et al., 2023).

## 2 Antibody discovery using *in silico* technology

Existing therapeutic antibodies for SARS-CoV-2 were discovered in laboratory through various approaches that involves *in vitro* technology. Hybridoma technology (Köhler and Milstein, 1975) and phage display (Smith and Petrenko, 1997) are employed to produce antibodies for SARS-CoV-2 with a wide range of application (Antipova et al., 2020; Kim et al., 2022; Somasundaram et al., 2020; Wang et al., 2023). Despite having many benefits to producing mAbs, *in vitro* technology poses limitations in terms of expenses as the methods mentioned above require sophisticated and resource-intensive high-throughput screening and characterization processes, which also consume adequate time (Moraes et al., 2021). In this case, *in silico* technology complements *in vitro* technology and can overtake several stages of conventional antibody discovery methods.

*In silico* antibody discovery comprises a multi-staged computational approach that accelerates the precision of antibody development. The process begins with the analysis of antibody sequences extracted from databases such as Protein Data Bank (PDB) (Bernstein et al., 1977), UniProt (UniProt Consortium, 2015) and other specified databases listed in Table 2. Modeling of 3D antibody structure is performed using predictive computational tools after sequence analysis to generate structural models with detailed spatial analysis. The next stage involves the evaluation of antibody interaction with targeted antigens through molecular docking. In this stage, high-affinity antibody candidates can be identified by predicting their interaction profiles. Finally, the developability of the antibodies will be evaluated via molecular dynamic simulation since the simulation refines the antibody-antigen complexes by examining their manufacturability. In recent times, *in silico* approach has been used widely in producing potential therapeutic options for COVID-19 through computational tools as presented in Table 1. *In silico* technology has been applied into SARS-CoV-2 antibody discovery in various stages of the process. Computational tools that can be used in different stages of SARS-CoV-2 antibody discovery are listed in Table 2.

**TABLE 1:** Overview of *in silico* technology application in producing potential therapeutic options for COVID-19.

Article	Findings through *in silico* approach	Stages of antibody discovery using *in silico* approach						
		Antibody sequence database and structural database	Analysis of antibody sequences	Modeling of 3D antibody	Visualization of 3D antibody	Evaluation of 3D antibody	Evaluation of antibody interaction	Molecular dynamic simulation
Su et al. (2024)	To investigate the relationship between point mutation in the upstream region of the HR2 Motif of S protein and the binding capacity of mAb-39			✓		✓	✓	✓
Gu et al. (2021)	To predict potential immunogenicity risk by accessing potential T-cell epitopes		✓					
Roodink et al. (2024)	To predict solvent-exposed, potential N-glycosylation site in the Framework 1 region of Ab 22-D9 (N20) and one in the Framework 3 region of Ab 21-F2 (N92)			✓		✓		
Alshahrani (2023)	To analyse the binding mechanisms and impact of Omicron mutations on different classes of antibodies targeting the SARS-CoV-2 RBD.	✓	✓		✓		✓	
Fung et al. (2022)	To investigate the binding stability of RBD variants targeting a number of convalescent antibodies	✓					✓	✓
Boorla et al. (2023)	To produce structure guided design of fully *de novo* high affinity antibodies against specific epitopes of SARS-CoV-2 spike protein	✓	✓	✓		✓	✓	
Cheng et al. (2020)	To study the interference of 6D3 with SARS-CoV-2 viral entry by competing with the host cell proteases	✓		✓		✓	✓	
Hernandez et al. (2023)	To provide a proof-of-concept study for the computational design of high-affinity antibodies that bind to multiple variants of the SARS-CoV-2 spike protein	✓	✓		✓	✓		
García-Vega et al. (2023)	To investigate the interaction of 19n01 with RBD in the Omicron BA.2, BA.3, and BA.4/5 subvariants			✓			✓	✓
Desautels et al. (2023)	To redesign and renew the efficacy of COV2-2130 against Omicron BA.1 and BA.1.1 strains while maintaining efficacy against the dominant Delta variant	✓	✓	✓			✓	✓
Schepens et al. (2021)	To enhance the affinity of broadly neutralizing VHH that can combat COVID-19 *in vivo*		✓	✓			✓	✓
Yu et al. (2022)	To enhance the binding affinity of the antibody	✓					✓	✓

This table summarizes research articles that incorporated *in silico* technology in various stages of SARS-CoV-2 antibody discovery process. Each reference listed under articles are individual research on in antibody discovery, followed by the finding of the research using *in silico* approach that highlights the key outcomes in the respective studies. Specific stages of antibody discovery where the computational approaches were used also identified in this table. For stages with *in silico* approach incorporation, a checkmark (✓) is used to indicate its inclusion. This table provide a comprehensive view of the diverse roles that *in silico* methods can play in antibody discovery and their adoption in different stages of the process.

**TABLE 2:** Computational tools used in different stages of antibody discovery *in silico.*

Application in *silico* technology	Tools	Usage	References
Databases	UniProt	Provides well-annotated protein sequences	UniProt Consortium (2015)
	Protein Data Bank (PDB)	A repository for biological macromolecular crystal structures	Bernstein et al. (1977)
	SwissProt database	Provides non-redundant protein sequences	Bairoch and Apweiler (2000)
	PROSITE	A protein data repository	Hulo et al. (2006)
	Structural Classification of Proteins (SCOP) database	Provide the most recent version of PDB of a protein	Lo Conte et al. (2000)
	Structural Antibody Database (SAbDab)	Provides antibody structural data	Dunbar et al. (2013)
	Therapeutic Structural Antibody Database (Thera-SAbDab)	Antibody sequence repository, after numbered and aligned all therapeutic variable domain sequences to the sequences of known structures in SAbDab	Raybould et al. (2020)
Antibody Sequence Analysis	Antibody region-specific alignment (AbRSA)	Determines CDR through numbering the sequence	Li et al. (2019)
	ANARCI	Annotates antibody and antigen receptor variable domain amino acid sequences from various species with different numbering schemes	Dunbar and Deane (2015)
3D Modeling of Antibody	SWISS-MODEL	Offers an automated modeling tool that is simple to use and incorporates expert knowledge, where the approach is characterized as rigid fragment assembly	Schwede et al. (2003)
	MODELLER	Offers modeling of comparative protein structures	Šali and Blundell (1993)
	AlphaFold2	Offers an extensive deep-learning framework for protein structure prediction	Skolnick et al. (2021), Ruff and Pappu (2021), and Cheung et al. (2023)
	RoseTTAFold	Model protein-protein complexes using only sequence information	Liang et al. (2022)
	ABodyBuilder	Model antibody only	Leem et al. (2016)
Visualize 3D Antibody Model	PyMOL	Visualise protein molecules in various representations	DeLano (2002)
	Visual Molecular Dynamics (VMD)	To view wider-ranging molecules including protein	Humphrey et al. (1996)
Evaluation of 3D Antibody Interaction	ClusPro	Permits the direct docking of two interacting proteins	Kozakov et al. (2017)
	High Ambiguity Driven Docking Approach (HADDOCK)	Docking tool that harness biochemical and biophysical interaction data	Dominguez et al. (2003)
	RosettaDock	Offers multi-scale docking approach that combines a high-resolution, all-atom refinement stage that optimizes both rigid-body orientations and side-chain conformation with a low-resolution, centroid-mode, and coarse-grain stage	Lyskov and Gray (2008)
	ZDOCK	A docking tool that uses Fast Fourier Transform (FFT) to optimize electrostatics, desolvation, and GSC score that defines the total number of grid points in this layer that overlap any grid points belonging to ligand atoms to yield less a clash penalty	Chen et al. (2003)
	HawkDock	A docking tool is developed by the HawkDock server with the integration of the ATTRACT docking algorithm and the MM/GBSA free energy	Weng et al. (2019)
Molecular Simulation of Antibody-antigen Complex	GROMACS (Groningen Machine for Chemical Simulations)	An open-source software package designed for molecular dynamics simulations of biochemical molecules including proteins	Berendsen et al. (1995) and Van Der Spoel et al. (2005)

This table outlines the key stages involves in *in silico* antibody discovery for SARS-CoV-2, along with the computational tools used at each stage, as described in the following sections of the review. The databases to acquire antibody and antigen sequences are also included in this table.

### 2.1 Analysis of antibody sequences

Sequences of antibody discovered as therapeutic option for COVID-19 are required to be analyzed before subjecting the sequence for further analysis. Since all variable domains fold into a series of beta strands joined by loops in a very similar 3D shape, the complementarity-determining regions (CDRs) are six of these loops at the top, where these regions develop loops that extend from the surface of the antibody, will result in direct contact with the antigen (Davies and Chacko, 1993). Numbering each residue according to a conventional approach is very helpful for sequence comparisons and engineering due to the continuity of the antibody structural similarity. Precise identification and characterization of these antibody regions are crucial in development and modification of antibodies (Patel et al., 2023). These annotated CDRs establish a significant degree of variation in antibody structure (Wong et al., 2019). Hence, it is critical for recognizing CDR to ensure its binding to a specific antigenic molecule before posing modifications to the antibody.

Numbering schemes with different approaches and set of applications have been developed to standardize the annotation of CDRs. An early yet widespread approach for annotating CDRs is the Kabat numbering scheme, which detects hypervariable regions and relies on the antibody sequences alignment (Kabat, 1991). The 3D structure of the antibodies is the foundation of Chothia numbering scheme (Chothia and Lesk, 1987) which emphasizes the structural locations of CDRs and the protected framework areas that sustain them. An enhanced version of the original Chothia scheme, the Martin scheme, introduces more structural insights and improves the numbering to cover a greater number of spots (Martin and Thornton, 1996), however, it has not been widely utilized. The well-established and comprehensive IMGT numbering scheme, annotates immunoglobulin and T cell receptors (Lefranc et al., 2015). It offers a standardized framework for comparing different species by ensuring consistency across species and antibody types by defining CDRs using both sequence and structural data.

Immunogenicity of the antibody sequences is also predicted to assess the immunogenic response of the therapeutic antibody which ensures safety and effectiveness. Immunogenicity prediction analysis helps in determining whether the antibody sequences exhibit low immunogenicity by identifying significant epitopes and ensuring that they fall below thresholds associated with strong immune activation. These antibodies can enhance their feasibility and reduce detrimental immune responses in various patient populations (Harris and Cohen, 2024).

ANARCI (Dunbar and Deane, 2015), an online tool that offers to annotate variable domains of antibodies from various species, enabling precise identification of CDRs and their alignment for immunogenicity analysis, is widely used in several SARS-CoV-2 studies (Wang et al., 2022; Xu et al., 2021; Zhou et al., 2023). Antibody region-specific alignment (AbRSA) (Li et al., 2019), is also a platform to perform sequence analysis by delimiting the CDRs and antibody numbering for numerous antibodies targeting viral particles (Dănăilă and Buiu, 2022; Dzimianski et al., 2023; Singh et al., 2023).

### 2.2 Modeling of 3D antibody

The successive unfolding process of protein folding transforms the protein sequences of the SARS-CoV-2 binding antibodies, which are mostly composed of a linear sequence of amino acids, into a functional three-dimensional antibody structure (Poluri et al., 2021). The arrangement of the amino acids determines its basic structure. From this linear arrangement, localized folding results in the formation of secondary structures including alpha helices and beta sheets, which are fuelled by hydrogen bonds between adjacent amino acids. The intricate three-dimensional tertiary structure is its repercussions of the continuous folding of the secondary structure together with the inclusion of loops and turns of the antibody (Rehman et al., 2022).

Protein folding analysis provides many useful insights about the interaction of the antibody especially through identifying the structure of CDR loop formations, but this multifaceted process requires expensive and specialized equipment, making it a challenging task before computational tools are being employed (Benjin and Ling, 2020; Brito and Archer, 2020). But as time passes, using *in silico* technology, where protein modeling has allowed for generally reliable predictions to be made (Srivastava et al., 2018). The goal of protein modeling is to make use of a range of computer methods to analyze amino acid sequences to predict the three-dimensional (3D) structure of the antibody sequences. Protein modeling provides distinctive approaches for predicting protein structures through a variety of tools that has been included on Table 2, which uses the protein sequences as an input (Agnihotry et al., 2022).

AlphaFold2 (Cheung et al., 2023; Ruff and Pappu, 2021; Skolnick et al., 2021) produces remarkably accurate 3D structure predictions using a neural network architecture that has been trained on a large database of structural and protein sequence data. This tool is utilized in various SARS-CoV-2-related studies that explore the binding behavior of its structural proteins (Ali and Caetano-Anollés, 2024; Jiao et al., 2023; Raisinghani et al., 2024). There are also several studies on the structural analysis of antibodies that prove the modeling capability of AlphaFold2 for antibody sequences (Du and Huang, 2023; Yin et al., 2022). SWISS-MODEL (Schwede et al., 2003) utilizes a homology-modeling approach that is performed iteratively until a satisfactory model structure is obtained. 3D structures of SARS-CoV-2 antibodies (Schepens et al., 2021; Beshnova et al., 2022) were successfully determined through this tool. MODELLER (Šali and Blundell, 1993) is a 3D modeling standalone tool, used to predict the 3D structure of SARS-CoV-2 antibodies (Mercurio et al., 2021; Yang et al., 2021) and restore missing residues in its structure (Martí et al., 2022; Giron et al., 2020). RoseTTAFold is one of the modeling tools that uses neural network-based techniques, incorporating connection between sequences, atomic coordinates, residue-residue orientations, and distances. This tool has been used in several studies on SARS-CoV-2 antibody discovery (Ford et al., 2022; Jing et al., 2024; Lubin et al., 2021). ABodyBuilder (Leem et al., 2016) is an antibody modeling software that incorporates multiple tools, including ABangle (Dunbar et al., 2013) and FREAD (Choi and Deane, 2010). Since this tool is specialized for antibody modeling, numerous studies employed ABodyBuilder to model the variable region of antibodies (Das et al., 2022; Das et al., 2023; Rouet et al., 2023) which also includes bispecific antibody (Ojha et al., 2022).

### 2.3 Evaluation of antibody interaction

The specificity of a novel or pre-existing antibody of SARS-CoV-2 can be accessed through validation *in silico* using computational tools. The binding properties of an antibody are primarily determined by the sequence and structure of CDRs through molecular docking. Molecular docking is performed using the analyzed and modeled 3D antibody structures to study the interaction by predicting the preferred orientation, affinity, and interaction of an antibody-antigen complex by analyzing intermolecular interactions (Koçer and Çelik, 2024).

Molecular docking is a process that anticipates atomic-level molecular interactions (Agu et al., 2023). Molecular docking can be performed with various types of biological molecules which include small molecules such as drugs, metabolites, ligands, inhibitors, ions (Jarad et al., 2023; Noreen et al., 2023), and complex molecules that comprise DNA, RNA, proteins, peptides, carbohydrates, nucleosides (Aziz et al., 2023; Madku et al., 2023; Weng et al., 2020). According to research by (Shahmirzaie et al., 2020), molecular docking has proven its capability of being a pioneering analysis to validate biological model interaction by providing binding site information. In the process of validation of antibody binding, molecular docking helps in predicting the preferred orientation of an antibody to the targeted antigen when these molecules are bound to each other to form a stable complex (Gaudreault et al., 2023).

Binding of an antibody exhibits both rigid and flexible properties which is essential for efficient antigen recognition and immune response (Fernández-Quintero et al., 2020). Electrostatic interactions and complementary structures lead to a relatively rigid and specific binding between the paratope and epitope where the rigidity ensures high-affinity binding and specificity (Zeng et al., 2023). On the other hand, the antibody also exhibits flexibility that facilitates conformational changes in the antigen and antibody. Flexibility allows the antibody to bind to a wide range of epitopes and identify antigens with various conformations by allowing it to accommodate variations in the antigen structure (Kilambi and Gray, 2017). An induced-fit mechanism takes place in binding conditions, where the conformational changes between the antigen and antibody are made upon binding to enhance their interactions. The flexibility of an antibody allows it to adapt to the structural alterations in the antigen and improves binding affinity (Bekker et al., 2020). In general, an antibody requires dynamic equilibrium between rigid and flexible phases upon its binding to the antigen.

RosettaDock is a docking approach that optimizes both rigid-body orientations and side-chain conformation (Lyskov and Gray, 2008). RosettaDock is used to perform docking of nanobodies against SARS-CoV-2 receptor-binding domain (RBD) (Yang et al., 2021), monoclonal antibodies against rare antigenic site of SARS-CoV-2 spike protein (Suryadevara et al., 2024) and a specific antibody against SARS-CoV-2 spike protein to improvise the binding affinity (Neamtu et al., 2023). ZDOCK uses Fast Fourier Transform (FFT) to yield less clash penalty in docking (Chen et al., 2003). Several studies employed ZDOCK to study the SARS-CoV-2 antibody-antigen interaction (Khan et al., 2020; Nath et al., 2021). HawkDock is an unique docking tool with integration of the ATTRACT docking algorithm and the MM/GBSA free energy that allows determination of antibody-antigen binding precisely (Weng et al., 2019). Docking is performed through this tool with nanobodies and therapeutic antibodies for interaction analysis (Shah and Woo, 2022; Yang et al., 2024). ClusPro is a widely used docking tool that has benchmarked against alternative docking tools in Critical Assessment of Predicted Interactions (CAPRI) studies (Kozakov et al., 2017). This tool employed to study the binding properties of SARS-CoV-2 spike protein RBD with nanobodies (Shang et al., 2024) and SARS-CoV-2 spike protein with monoclonal antibodies (Nath et al., 2021). High Ambiguity Driven Docking Approach (HADDOCK) harnesses biochemical and biophysical interaction data, including mutagenesis or chemical shift perturbation data from NMR titration experiments to obtain near-native results. Binding prediction of the antibodies discovered with the targeted site on SARS-CoV-2 is performed in several studies using this tool (Ford et al., 2022; Ford et al., 2023).

### 2.4 Developability evaluation of antibody

The developability of antibody models discovered using *in silico* approach for COVID-19 will be studied and validated as they can aligned with the real-time experimentally produced therapeutic antibodies. Molecular dynamics (MD) simulations offer a dynamic and comprehensive understanding of biomolecular behavior at the atomic level, and have developed to be an essential tool in the study of computational biophysics (Lemm et al., 2021). In the field of antibody design, MD simulations have shown to be very helpful as a reliable means of testing *in silico* designs, bridging the gap between computational predictions and experimental findings by providing insights into the structures.

MD simulations operate based on the basic principles of classical mechanics, which make use of Newton’s equations of motion to predict the motions of individual atoms in a molecular system (Shukla and Tripathi, 2020). MD simulations accurately depict the interactions between atoms, including the flexibility of bonds, angle bending, and non-bonded interactions such as van der Waals forces and electrostatics, by applying a force field, a mathematical model that defines the potential energy of the system (Badar et al., 2022). The force field selection is essential to the precision and dependability of MD simulations since it significantly impacts the simulation outcomes. Numerous force fields with unique strengths and applications have been developed over time. CHARMM force field is one of the most common and extensible force fields in computational chemistry which operates exceptionally well to simulate lipids, proteins, and nucleic acids (Brooks et al., 2009). AMBER force field is particularly utilized for proteins and nucleic acids (Wang et al., 2004). The goal of AMBER is to supply precise parameter sets for biomolecular systems. The temporary conformational state of antibody binding is not always visible in static crystal structures but only can be revealed by MD simulations. Accurate parameterization of these forcefields in MD simulation play pivotal roles in comprehending the principles underlying antibody binding and refining antibody architectures to enhance their affinity and specificity for target antigens (Shaw et al., 2010).

GROMACS, an open-source software package designed for molecular dynamics simulations of biochemical molecules including proteins, acts as an *in silico* to study the behavior of antibody and antibody-antigen complexes at the atomic level (Berendsen et al., 1995; Van Der Spoel et al., 2005). The stability of various SARS-CoV-2 antibody-antigen complexes, including complexes involving the SARS-CoV-2 S protein and bispecific antibodies, as well as the SARS-CoV-2 S protein trimer with monoclonal antibodies, was assessed by measuring the root-mean-square fluctuation (RMSF) of the complexes to quantify dynamic stability (Ford et al., 2022; Ford et al., 2023).

## 3 Discussion

The global response to the SARS-CoV-2 outbreak has emphasized the critical necessity of quick therapeutic progress. Handling SARS-CoV-2 live virus necessitates adherence to Biosafety Level 3 (BSL-3) laboratory standards as SARS-CoV-2 can be transmitted by air that can lead to respiratory transmission (Kaufer et al., 2020). Compliance with the biosafety regulations of BSL-3 adds to the time and cost of research as it requires a list of facilities and personal protective equipment (Loibner et al., 2021). In this case, *in silico* approach have grown to be valuable in antibody discovery of SARS-CoV-2.

The usage of computational tools complements various parts of the experimental approach of antibody discovery for SARS-CoV-2. The process of discovering new antibodies necessitates creating antibody libraries consisting of a pool of antibodies featuring various binding sites and screening them to select the antibody candidates with the best binding affinities (Kelley, 2020). Thus, the usage of molecular docking streamlines the process by cutting down the necessity to use experimental approach, which includes handling SARS-CoV-2 antigen or virus for repeated screening (Alshahrani, 2023; Boorla et al., 2023; Gaudreault et al., 2023).

Molecular dynamic simulation bridges the gap between the *in silico*-developed antibodies and experimentally produced antibodies by mimicking the near-native condition of the antibody (Jandova et al., 2021). Researchers can minimize the repeated usage of live SARS-CoV-2 virus and other experimental assays as these simulations reduce the dependence on experimental assessments while retaining a high level of accuracy (Jairajpuri et al., 2021). Determination of antibody 3D structure is also one of the most essential contributions of *in silico* approach in antibody discovery. 3D modelling is a useful complement to approaches such as cryo-electron microscopy (cryo-EM) and X-ray crystallography for predicting the three-dimensional structure of antibodies. Computational modeling of 3D structure of the antibodies offers a cost-effective alternative, as the equipment required for the conventional approach is expensive to acquire and maintain (Benjin and Ling, 2020; Brito and Archer, 2020).

Although implementation of *in silico* approach in SARS-CoV-2 antibody discovery significantly reduce the time and resource investments, transitioning from *in silico* predictions to experimentally validated antibodies present a few limitations. Biological systems are inherently complex, and *in silico* models often oversimplify these intricacies. Although *in silico* approaches can predict the near-native structure and conditions of antibodies, it unable to capture the complexity of the biological system such as glycosylation (Kashkooli et al., 2021). Hence, developing integrated workflows that combine *in silico* predictions with experimental validation can optimise the transition between these stages.

Moreover, the effectiveness of *in silico* tools heavily depends on the availability of high-quality training data. Rapid evolution of SARS-CoV-2 has resulted in limited repositories of updated experimentally validated sequences and structural data in public databases (Chen et al., 2022). Limited availability of the information may hinder the accuracy of the computational tools and the accuracy of the computational predicts is compromised by this shortage of data. Expansion of these databases and providing quality training datasets for computational tools are critical steps that enhance the performance of *in silico* tools (Norman et al., 2019; Khuat et al., 2024).

## 4 Conclusion

Antibody development is anticipated to accelerate at the greatest pace in upcoming years in life sciences, particularly in the fight against infectious diseases such as SARS-CoV-2. Researchers will be able to construct antibodies precisely but effortlessly due to the developments in bioinformatics and computer modeling. The *in silico* approach simplifies the process of antibody structure prediction and interaction analysis by providing a molecular dynamic simulation approach for validation. This method greatly improves the speed, economic performance, as well as effectiveness of the process of developing novel therapeutic antibodies. Although precision of computational assessments is reliant upon existing data and models, *in silico* technologies offer a quick and efficient means of prevention and treatment, that significantly reduce the worldwide burden of this infectious disease. The approaches are also having potential to resurface our knowledge of the immune system and antigen-antibody interaction advances. Overall, the idea of creating antibodies through *in silico* design has huge implications for the future prevention and management of SARS-CoV-2 and other infectious diseases.
